# Therapie des nicht-fernmetastasierten CRPC: Androgenrezeptorinhibition als neuer Therapiestandard beim nicht-fernmetastasierten kastrationsresistenten Prostatakarzinom mit hohem Metastasierungsrisiko

**DOI:** 10.1007/s00120-021-01473-0

**Published:** 2021-02-11

**Authors:** Boris Hadaschik, Eva Hellmis

**Affiliations:** 1 Urologische Universitätsklinik, Universitätsklinikum Essen, Hufelandstraße 55, 45147 Essen, Deutschland; 2 Urologicum Duisburg, Duisburg, Deutschland

**Keywords:** Prostatakrebs, Hormonentzugstherapie, Apalutamid, Darolutamid, Enzalutamid, Prostate cancer, Androgen deprivation therapy, Apalutamide, Darolutamide, Enzalutamlide

## Abstract

Bei Patienten mit nicht-fernmetastasiertem kastrationsresistentem Prostatakarzinom (nmCRPC oder M0CRPC) und hohem Progressionsrisiko (definiert als PSA-Verdopplungszeit ≤ 10 Monate) stellen selektive Androgenrezeptorinhibitoren (ARI) der neuen Generation in Kombination mit fortgeführter Androgendeprivationstherapie (ADT) den neuen Therapiestandard dar. Zugelassen sind derzeit Apalutamid, Enzalutamid und Darolutamid, die im Vorfeld bereits in den jeweiligen großen Phase-III-Zulassungsstudien SPARTAN, PROSPER und ARAMIS ihren primären Endpunkt metastasenfreies Überleben (MFS) erreicht hatten und jetzt nach längerer Nachbeobachtungszeit auch einen statistisch signifikanten und klinisch relevanten Gesamtüberlebensvorteil gegenüber Placebo plus ADT zeigen konnten. Die bisherige Datenlage weist auf eine vergleichbar gute Effektivität aller drei Substanzen bei insgesamt guter Verträglichkeit hin. Auch die generell gute Lebensqualität dieser Patientenpopulation, die in der Regel noch keine tumorbedingten Symptome aufweist, konnte erhalten werden. Direkt vergleichende Head-to-head-Studien der drei zugelassenen Substanzen liegen bislang nicht vor.

## Abstract (en)

In patients with nonmetastatic castration-resistant prostate cancer (nmCRPC or M0CRPC) at high risk of progression (defined as prostate-specific antigen [PSA] doubling time ≤ 10 months), new androgen receptor inhibitors (ARI) in combination with continued androgen deprivation therapy (ADT) are considered the new standard of care. Apalutamide, enzalutamide, and darolutamide have been approved on the basis of improved metastasis-free survival (MFS) in the respective large pivotal studies SPARTAN, PROSPER and ARAMIS and now, with a longer follow-up period, were able to show also a statistically significant and clinically relevant overall survival advantage compared to placebo plus ADT. The data available to date indicate that all three ARIs are comparably effective, accompanied by good tolerability. Moreover, the generally good quality of life of this patient population, who usually has no tumor-related symptoms, was maintained. Comparative head-to-head trials of the three approved substances are not available yet.

## Hintergrund

Ein nicht-fernmetastasiertes kastrationsresistentes Prostatakarzinom (M0CRPC) liegt vor, wenn unter klassischer Androgendeprivationstherapie (ADT) trotz eines Serumtestosteronspiegels auf Kastrationsniveau (<50 ng/dl) ein biochemisches Rezidiv mit PSA-Werten ≥ 2 ng/ml auftritt und in der konventionellen Bildgebung (Knochenszintigraphie und CT) keine Fernmetastasen nachweisbar sind [1]. Dabei haben Patienten mit einer kurzen PSA-Verdopplungszeit ≤ 10 Monate ein signifikant erhöhtes Risiko, Fernmetastasen zu entwickeln und am Prostatakarzinom zu versterben [2, 3]. Für M0CRPC-Patienten bestand lange Zeit nur die Möglichkeit, die ADT trotz des biochemischen Rezidivs auch im Falle kurzer PSA-Verdopplungszeiten bis zum Nachweis von Fernmetastasen fortzuführen, um dann mit einer zugelassenen mCRPC-Therapie behandeln zu können. Für viele Betroffene war dieses abwartende Vorgehen zusätzlich zum erwähnten höheren Risiko für die Entstehung von Fernmetastasen und zu versterben psychisch sehr belastend [4]. Denn ist einmal das Stadium des mCRPC mit einer vorliegenden Metastasierung erreicht, geht dies mit einer zunehmenden Symptomatik und einer deutlich schlechteren Überlebensprognose einher [5]. Vor allem Knochenmetastasen sind oft mit Schmerzen, pathologischen Frakturen und Rückenmarkskompression verbunden, was die Lebensqualität der Betroffenen erheblich beeinträchtigt [5]. Daher sollte bei M0CRPC-Patienten der Progress zum mCRPC möglichst lange verhindert werden. Die Verzögerung des Auftretens von Fernmetastasen (M1) war in allen aktuellen Zulassungsverfahren beim M0CRPC der primäre Endpunkt (metastasenfreies Überleben [MFS]; [6–8]). Das MFS, definiert als Dauer bis zum ersten radiologischen Nachweis von Fernmetastasen oder Tod, korrelierte in früheren Studien beim lokal begrenzten Prostatakarzinom [9] und jetzt auch in den Studien beim M0CRPC mit dem Gesamtüberleben (OS; [10–12]). Da die Patienten im nicht-metastasierten Stadium in der Regel noch asymptomatisch sind und der Allgemeinzustand noch wenig beeinträchtigt ist, stellt auch der möglichst lange Erhalt der gesundheitsbezogenen Lebensqualität ein wichtiges Therapieziel dar.

Mit Apalutamid, Darolutamid und Enzalutamid sind aktuell drei selektive ARI zugelassen, die jeweils in Kombination mit fortgeführter ADT den neuen Standard beim Hochrisiko-M0CRPC darstellen und die auch schon in den bereits aktualisierten Leitlinien der European Association of Urology (EAU) und des National Comprehensive Cancer Networks (NCCN) von 2020 mit einer starken Empfehlung (EAU) bzw. als Kategorie-1-Behandlungsoption (NCCN) empfohlen werden [13, 14]. Apalutamid ist in Europa seit Januar 2019 auf Grundlage der SPARTAN-Studie [6] in dieser Indikation zugelassen und erhielt zudem im Januar 2020 auf Basis der TITAN-Studie [15] die Zulassungserweiterung in Kombination mit ADT für die Behandlung des metastasierten hormonsensiblen Prostatakarzinoms (mHSPC). Enzalutamid und Darolutamid sind in Europa seit Oktober 2018 auf Basis der PROSPER-Studie bzw. seit März 2020 auf Basis der ARAMIS-Studie zur Behandlung des Hochrisiko-M0CRPC zugelassen [7, 8]. Alle drei Zulassungsstudien schlossen ein ähnliches Patientenkollektiv ein, hatten im Vorfeld jeweils ihren primären Endpunkt MFS erreicht und zeigten jetzt auch in den beim virtuellen ASCO 2020 präsentierten finalen Analysen eine signifikante OS-Verlängerung [12, 16, 17].

## Apalutamid beim Hochrisiko-M0CRPC

Apalutamid gehört ebenso wie Enzalutamid und Darolutamid zur neuen Generation selektiver ARI und weist im präklinischen Maus-Xenotransplantat-Modell im Vergleich zu konventionellen Antiandrogenen wie Bicalutamid eine höhere Affinität zum Androgenrezeptor (AR) auf [18, 19]. Apalutamid bindet kompetitiv mit hoher Selektivität an den AR und greift auf mehreren Ebenen in die AR-Signalkaskade ein: Verhinderung des Andockens von Androgenen an den AR, Hemmung der Translokation des AR in den Zellkern und dessen Bindung an die DNA sowie Verhinderung der AR-vermittelten Transkription [18, 19].

Nach den positiven Ergebnissen einer Phase-II-Studie mit 51 M0CRPC-Patienten mit hohem Metastasierungsrisiko [20] folgte die Phase-III-Zulassungsstudie SPARTAN, in der 1207 Patienten mit Hochrisiko-M0CRPC 2:1 randomisiert zusätzlich zur laufenden ADT Apalutamid 240 mg täglich (*n* = 806) oder Placebo (*n* = 401) erhielten [6]. Die Patienten waren bei Einschluss durchschnittlich 74 Jahre alt und wiesen eine mediane PSA-Verdopplungszeit von weniger als 5 Monaten auf. Positive Lymphknoten im kleinen Becken < 2 cm unter der iliakalen Bifurkation (N1-Stadium) waren erlaubt und bei rund 16 % der Patienten vorhanden.

**Figure 1:**
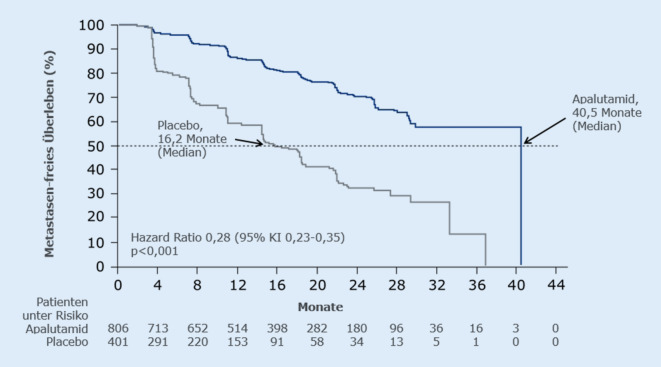

In der ersten geplanten Zwischenanalyse nach Erreichen von 378 MFS-Events (medianer Follow-up 20,3 Monate) war das mediane MFS im Apalutamid-Arm mit 40,5 Monaten vs. 16,2 Monaten im Placeboarm signifikant um mehr als 2 Jahre (24,3 Monate) verlängert und das Risiko für Metastasen oder Tod um 72 % verringert (HR 0,28; *p* < 0,001; Abb. 1; [6]). Dieser Vorteil war in allen untersuchten Subgruppen konsistent. Die Studie wurde daraufhin entblindet und ein Cross-over in den Apalutamid-Arm ermöglicht, was 76 Patienten (19 %) der Placebogruppe nutzten. Signifikant verbessert waren auch die sekundären Studienendpunkte: mediane Zeit bis zur symptomatischen Progression, mediane Zeit bis zur Metastasierung und medianes PFS (alle *p* < 0,001; [6]). Für das OS lagen zum Zeitpunkt der primären MFS-Analyse noch keine finalen Daten vor [6].

**Figure 2:**
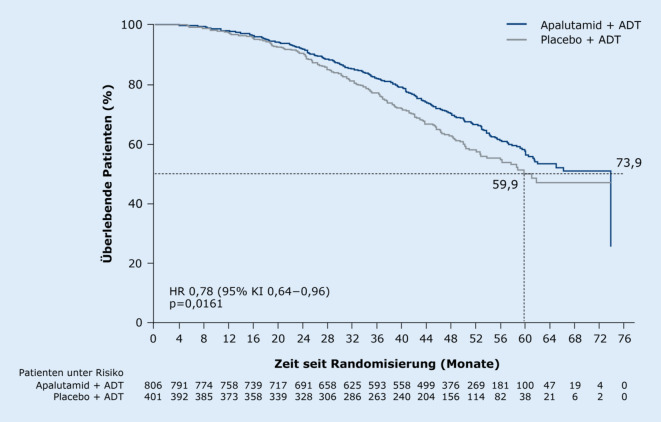

**Figure 3:**
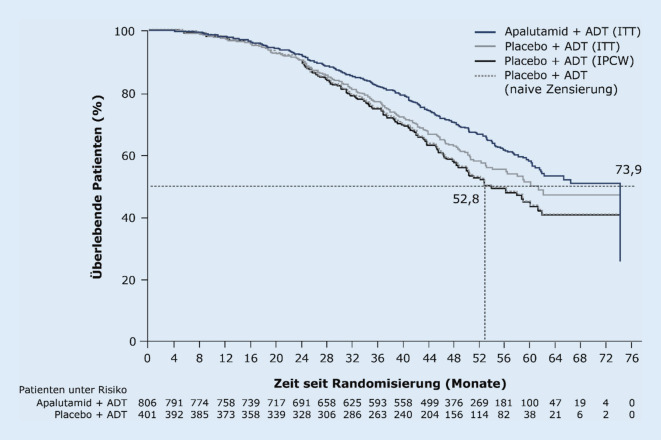

Die beim ASCO 2020 präsentierte und bereits auch publizierte finale Analyse der SPARTAN-Studie belegte für Apalutamid jetzt auch einen statistisch signifikanten OS-Vorteil: Bei einem medianen Follow-up von 52,0 Monaten (Datenschnitt 1. Dezember 2019) war das mediane OS signifikant um 14 Monate gegenüber Placebo verlängert (73,9 vs. 59,9 Monate) und das Sterberisiko um 22 % vermindert (HR 0,78; *p* = 0,016; Abb. 2; [10, 16]). Dieser signifikante OS-Vorteil konnte erreicht werden, obwohl mehr Patienten im Placeboarm nach dem Progress weitere lebensverlängernde Therapien erhalten haben (71 vs. 48 %) und zudem 76 Placebopatienten (19 %) nach Entblindung der Studie auch mit Apalutamid für median 26,1 Monate behandelt wurden (Cross-over-Gruppe; [10]). Die Gesamtrate an Patienten mit einer lebensverlängernden Folgetherapie betrug 84 % (Placeboarm) vs. 48 % (Apalutamid-Arm), was vermutlich den Effekt auf das OS zuungunsten von Apalutamid verzerrt haben dürfte. Darauf deuten die für Cross-over korrigierten Sensitivitätsanalysen mit dem IPCW(„inverse probability of censoring weighting“)-Verfahren und mit naiven Zensieren hin, die praktisch deckungsgleich eine OS-Differenz von 21,1 Monaten für mit Apalutamid behandelte Patienten gegenüber Placebo zeigten (73,9 vs. 52,8 Monate; Abb. 3; [10]).

Die Analyse des zweiten progressionsfreien Überlebens (PFS2, explorativer Endpunkt), das als Zeit zwischen Randomisierung und Krankheitsprogression oder Tod unter der zweiten Therapielinie (nachfolgende mCRPC-Erstlinientherapie) definiert war und speziell nur in der SPARTAN-Studie erhoben wurde, weist darauf hin, dass die Vorteile einer frühen Therapie nicht durch eine nachfolgende potente mCRPC-Therapie ausgeglichen werden können [6]. Ergänzende explorative Biomarkeranalysen der SPARTAN-Studie zeigten, dass im Apalutamid-Arm wenig nachweisbare resistenzvermittelnde AR-Anomalien unter Therapie auftraten [21]. In diesem Zusammenhang wurde bisher angenommen, dass die AR-Splice-Variante V7 (AR-V7) nicht nur zu einer Resistenz gegenüber hormonellen Therapien beiträgt, sondern auch mit einem nachteilhaften klinischen Resultat assoziiert ist [22]. Eine aktuelle Studie von Erb et al. zeigte jedoch keinen signifikanten Unterschied im PFS zwischen AR-V7-positiven und -negativen mCRPC-Patienten [23].

Apalutamid wurde in der SPARTAN-Studie insgesamt gut vertragen. Tab. 1 zeigt die Nebenwirkungsraten sowie die Raten der nebenwirkungsbedingten Therapieabbrüche und Todesfälle. Häufiger unter Apalutamid und meist vom Grad 1–2 waren Fatigue (30,4 vs. 21,1 %), Hautausschläge (23,8 vs. 5,5 %), Arthralgie (15,9 vs. 7,5 %), Gewichtsverlust (16,1 vs. 6,3 %), Stürze (15,6 vs. 9,0 %), Knochenfrakturen (11,7 vs. 6,5 %) und Hypothyreose (8,1 vs. 2,0 %; [6]). Die häufigsten Grad-3- bis -4-Nebenwirkungen unter Apalutamid waren Hautausschläge (5,2 vs. 0,3 %), Frakturen (2,7 vs. 0,8 %) und Stürze (1,7 vs. 0,8 %; [6]). Die Hautausschläge waren in den meisten Fällen leicht ausgeprägt, vorübergehend und mit Supportivmaßnahmen, Therapieunterbrechungen und/oder Dosisreduktionen gut handhabbar und führten im Apalutamid-Arm nur in 2,4 % der Fälle zum Studienabbruch ([6], Tabelle S4 im Supplement). Das Sicherheitsprofil war in der finalen Analyse konsistent mit den vorherigen Ergebnissen [10]. Die mit den krankheitsbezogenen Fragebögen FACT-*P* und EQ-5D-3L erhobene Lebensqualität blieb im Apalutamid-Arm erhalten, während es im Placeboarm nach ca. einem Jahr zur Verschlechterung kam [24, 25]. Eine Abnahme der Lebensqualität war mit einer symptomatischen Progression der Erkrankung verbunden [24, 25].

**Table 1:** 

	Apalutamid + ADT (*n* = 803)	Placebo + ADT (*n* = 398)
Follow-up, median (Monate)	52,0	52,0
Behandlungsdauer, median (Monate)	32,9	11,5
UE gesamt (%)	97,3	93,7
Schwere UE, Grad 3–4 (%)	55,9	36,4
Schwerwiegende UE (%)	36,1	24,9
Therapieabbruch wegen UE (%)	14,9	7,3
UE mit Todesfolge (%)	3,0	0,5

*ADT* Androgendeprivationstherapie, *UE* unerwünschte Ereignisse (Nebenwirkungen)

## Enzalutamid beim Hochrisiko-M0CRPC

In der PROSPER-Studie verlängerte Enzalutamid plus ADT vs. Placebo plus ADT das mediane MFS signifikant um 21,9 Monate, von 14,7 auf 36,6 Monate (HR 0,29; *p* < 0,001; [7]). Damit war das Risiko für Metastasen oder Tod um 71 % reduziert. An der Studie nahmen insgesamt 1401 Hochrisiko-M0CRPC-Patienten teil und erhielten 2:1 randomisiert zusätzlich zur fortgeführten ADT einmal täglich 160 mg Enzalutamid (*n* = 993) oder Placebo (*n* = 468). Die sekundären Endpunkte Zeit bis zur PSA-Progression und Zeit bis zur nächsten antineoplastischen Therapie waren ebenfalls signifikant verbessert (alle *p* < 0,001). Mit der finalen Analyse der PROSPER-Studie (medianer Follow-up 48,0 Monate) konnte auch für Enzalutamid eine signifikante Reduktion des Sterberisikos um 27 % mit einer Verlängerung des medianen OS um 10,7 Monate gezeigt werden (67,0 vs. 56,3 Monate; HR 0,73; *p* = 0,001; [11, 17]). Auch die PROSPER-Studie wurde nach der primären MFS-Analyse entblindet. Die Cross-over-Rate betrug hier 18,7 % (*n* = 87) und die mediane Behandlungsdauer der Cross-over-Gruppe mit Enzalutamid 14,5 Monate. Weitere lebensverlängernde Post-Progressionstherapien erhielten 65 % der Placebogruppe (darunter 59 % Abirateronacetat, 47 % Docetaxel) vs. 33 % der Enzalutamid-Gruppe (darunter 60 % Docetaxel, 49 % Abirateronacetat).

Die Nebenwirkungsraten sowie die Raten der nebenwirkungsbedingten Abbrüche und Todesfälle finden sich in Tab. 2. Die häufigsten Nebenwirkungen unter Enzalutamid waren Fatigue (33 vs. 14 %), Stürze (17 vs. 8 %), Hypertonie (12 vs. 5 %), kardiovaskuläre Ereignisse (5 vs. 3 %) sowie Konzentrations- und Gedächtnisstörungen (5 vs. 2 %; [11]). Die nebenwirkungsbedingte Abbruchrate betrug 17,0 % (Enzalutamid) vs. 9,0 % (Placebo). Unter Enzalutamid plus ADT zeigte sich keine Verschlechterung der Lebensqualität gegenüber Placebo plus ADT [11].

**Table 2:** 

	Enzalutamid + ADT (*n* = 930)	Placebo + ADT (*n* = 465)
Follow-up, median (Monate)	48	48
Behandlungsdauer, median (Monate)	33,9	14,2
UE gesamt (%)	94,0	82,0
Schwere UE, Grad 3–4 (%)	48,0	27,0
Schwerwiegende UE (%)	40,0	22,0
Therapieabbruch wegen UE (%)	17,0	9,0
UE mit Todesfolge (%)	5,0	1,0

*ADT* Androgendeprivationstherapie, *UE* unerwünschte Ereignisse (Nebenwirkungen)

## Darolutamid beim Hochrisiko-M0CRPC

Die ARAMIS-Studie umfasste insgesamt 1509 Patienten mit Hochrisiko-M0CRPC, von denen 955 Patienten 2‑mal täglich 600 mg Darolutamid mit Nahrung erhielten und 554 erhielten Placebo [8]. Im Gegensatz zu SPARTAN und PROSPER waren in ARAMIS die PSA-Werte nicht verblindet und somit den Ärzten und Patienten während des Studienverlaufes bekannt. Mit einem medianen Follow-up von 17,9 Monaten führte Darolutamid gegenüber Placebo zu einer signifikanten Verlängerung des medianen MFS von 18,4 auf 40,4 Monate (HR 0,41; *p* < 0,001; [8]). Darolutamid verbesserte auch die sekundären Endpunkte Zeit bis zur Schmerzprogression (*p* < 0,001), Zeit bis zum ersten symptomatischen Skelettereignis (*p* = 0,005) und Zeit bis zur Chemotherapie (*p* < 0,001) signifikant [8]. In der aktuellen finalen Analyse (medianer Follow-up 29 Monate) war das Sterberisiko im Darolutamid-Arm signifikant um 31 % reduziert (HR 0,69; *p* = 0,003), wobei der Median in beiden Studienarmen noch nicht erreicht war [26]. Die 3‑Jahres-OS-Rate lag bei 83 vs. 77 %. Die Cross-over-Rate betrug in der ARAMIS-Studie 31 % (*n* = 170) und die mediane Behandlungsdauer der Cross-over-Gruppe mit Darolutamid 11 Monate [26]. Eine lebensverlängernde Folgetherapie nach Progress erhielten 56 % im Placeboarm vs. 15 % im Darolutamid-Arm [26].

Darolutamid war insgesamt sehr gut verträglich. Die meisten Nebenwirkungen waren von Grad 1 oder 2. Die Nebenwirkungsraten sowie die Raten der nebenwirkungsbedingten Abbrüche und Todesfälle sind in Tab. 3 aufgeführt. Eine wesentliche Zunahme von ARI-assoziierten Nebenwirkungen wie Stürzen, Frakturen, kognitiven Störungen, Krampfanfällen oder Hypertonie wurde nicht beobachtet. Häufigere Nebenwirkungen waren Fatigue (13,2 vs. 8,3 %; Grad 3–4: 0,4 vs. 0,9 %), Hypertonie (7,8 vs. 6,5 %; 3,5 vs. 2,3 %), Herzrhythmusstörungen (7,3 vs. 4,3 %; 1,8 vs. 0,7 %), Knochenbrüche (5,5 vs. 3,6 %; 1,0 vs. 0,9 %) und Stürze (5,2 vs. 3,6 %; 0,9 vs. 0,7 %; [26]). Die Lebensqualität blieb auch in der ARAMIS-Studie erhalten; die Zeit bis zur Schmerzprogression und bis zur Verschlechterung von Harnwegsbeschwerden war im Darolutamid-Arm länger [8].

**Table 3:** 

	Darolutamid + ADT (*n* = 954)	Placebo + ADT (*n* = 554)
Follow-up, median (Monate)	29,1	29,1
Behandlungsdauer, median (Monate)	25,8	11,6
UE gesamt (%)	86	79
Schwere UE, Grad 3–4 (%)	26	22
Schwerwiegende UE (%)	26	22
Therapieabbruch wegen UE (%)	9	9
UE mit Todesfolge (%)	4	3

*ADT*: Androgendeprivationstherapie, *UE* unerwünschte Ereignisse (Nebenwirkungen)

## Diskussion

Alle drei ARI der neuen Generation (Apalutamid, Enzalutamid und Darolutamid) zeigten in den Zulassungsstudien zum Hochrisiko-M0CRPC einen beeindruckenden onkologischen Benefit. Dabei kann aufgrund der aktuellen Datenlage hinsichtlich des MFS und OS insgesamt von einer vergleichbaren Effektivität aller drei Substanzen ausgegangen werden. Bei ähnlichen Patientenkollektiven zeigte sich eine MFS-Verlängerung um etwa 2 Jahre gegenüber Placebo, mit HR von 0,28 in SPARTAN, 0,29 in PROSPER und 0,41 in ARAMIS [6–8]. Mit den finalen Analysen aller 3 Studien zeigte sich aktuell auch eine signifikante Verlängerung der Gesamtüberlebenszeit um etwa ein Jahr mit entsprechenden HR von 0,78, 0,73 und 0,69 [10, 11, 26]. Bei der Bewertung der OS-Ergebnisse ist zu berücksichtigen, dass alle 3 Studien nach der primären MFS-Analyse entblindet wurden und ein Cross-over vom Kontroll- in den Verumarm erfolgte. Eine separate Analyse unter Berücksichtigung des Cross-over liegt nur für SPARTAN vor und zeigt eine deutliche Zunahme des OS-Vorteils im Vergleich zum Placeboarm (Abb. 3; [10]).

Im Hinblick auf die OS-Daten ist außerdem erfreulich und wichtig zu wissen, dass zu den aktuellen Analysezeitpunkten deutlich mehr Patienten in den Kontrollarmen aller Studien aufgrund einer Progression zum mCRPC weitere lebensverlängernde Therapien erhielten. Trotz dessen zeigte die frühe Therapie deutliche OS-Vorteile. Ein Vergleich der 3 Zulassungsstudien ist aus folgenden Gründen schwierig: Die finale ARAMIS-Analyse ist bei einem kürzeren Follow-up (median 29,1 Monate) und daher relativ unreifem OS-Datensatz (Ereignisrate: 15,5 % im Darolutamid-Arm vs. 19,1 % im Placeboarm) und deutlich geringerer Rate an lebensverlängernden Folgetherapien im Placeboarm erfolgt [26]; unterschiedliche Cross-over-Raten und unterschiedlich lange Behandlungsdauern der Cross-over-Gruppen mit der jeweiligen Substanz (median 26 Monate in SPARTAN, 14,5 Monate in PROSPER und 11 Monate in ARAMIS) müssen berücksichtigt werden [10, 11, 26].

Mit den 3 Studien wurde eine gute Korrelation des MFS in der konventionellen Bildgebung mit dem OS gezeigt, sodass in zukünftigen Studien das metastasenfreie Überleben in der konventionellen Bildgebung als früher Endpunkt validiert eingesetzt werden kann.

Hinsichtlich der Nebenwirkungen wiesen alle drei Substanzen generell ein sehr günstiges Sicherheitsprofil auf. Bei noch fehlenden direkten Head-to-head-Vergleichen ist aber auch bei der diesbezüglichen Interpretation der Daten Vorsicht geboten. Zu beachten sind beispielsweise Unterschiede der Intervalle der Sicherheitserhebung (alle 4 Wochen in SPARTAN vs. alle 16 Wochen in PROSPER und ARAMIS), der Studiendurchführung bzgl. Verblindung der PSA-Werte und der Behandlungsdauer. So betrug die mediane Behandlungsdauer in den vorgestellten Analysen bei Apalutamid 32,9 Monate, bei Enzalutamid 33,9 Monate und bei Darolutamid 25,8 Monate [10, 11, 26]. Innerhalb der einzelnen Studien war gegenüber dem jeweiligen Placeboarm die Behandlungsdauer im Apalutamid-Arm 2,9-mal länger (32,9 vs. 11,5 Monate), im Enzalutamid-Arm 2,4-mal länger (33,9 vs. 14,2 Monate) und im Darolutamid-Arm 2,2-mal länger (25,8 vs. 11,6 Monate; [10, 11, 26]). Darüber hinaus liegen für Darolutamid bisher nur Sicherheitsdaten aus der randomisierten Doppelblindphase sowie zur Cross-over-Gruppe aus der unverblindeten (Open-label‑)Phase, aber noch keine Langzeitdaten vor [26]. Schließlich sei noch darauf hingewiesen, dass trotz der Unterschiede im Nebenwirkungsprofil und bei den Abbruchraten die Lebensqualität in allen 3 Studien während der Behandlung erhalten blieb [6–8].

## Fazit für die Praxis

- Mit Apalutamid, Enzalutamid und Darolutamid stehen mittlerweile drei hochwirksame Therapieoptionen für das Hochrisiko-M0CRPC (nicht-fernmetastasiertes kastrationsresistentes Prostatakarzinom) zur Verfügung, die gleichwertig in den aktuellen internationalen Leitlinien empfohlen werden.
- Die durch die intensivierte Therapie deutlich verlängerte Gesamtüberlebenszeit der betroffenen Männer geht erfreulicherweise mit einer länger erhaltenen hohen Lebensqualität einher.
